# AI-based visualization of loose connective tissue as a dissectable layer in gastrointestinal surgery

**DOI:** 10.1038/s41598-024-84044-5

**Published:** 2025-01-02

**Authors:** Yuta Kumazu, Nao Kobayashi, Seigo Senya, Yuya Negishi, Kazuya Kinoshita, Yudai Fukui, Kazuhito Mita, Tomohiko Osaragi, Toshihiro Misumi, Hisashi Shinohara

**Affiliations:** 1 Anaut Inc., 2-1-6-19F WeWork Hibiya Park Front, Uchisaiwaicho, Chiyodaku, Tokyo, 100-0011 Japan; 2 Department of Surgery, Yokohama City University, Kanagawa, Japan; 3 Department of Surgery, Tsudanuma Central General Hospital, Chiba, Japan; 4 Department of Frontier Surgery, Graduate School of Medicine, Chiba University, Chiba, Japan; 5 Department of Gastroenterological Surgery, Toranomon Hospital, Tokyo, Japan; 6 Department of Surgery, Hadano Red Cross Hospital, Kanagawa, Japan; 7 Department of Data Science, National Cancer Center Hospital East, Chiba, Japan; 8 Department of Gastroenterological Surgery, Hyogo Medical University, Hyogo, Japan

**Keywords:** Gastrectomy, Colorectal surgery, Inguinal hernia repair, AI, Dissectable layer, Loose connective tissue, Gastroenterology, Medical research, Surgical oncology

## Abstract

We aimed to develop an AI model that recognizes and displays loose connective tissue as a dissectable layer in real-time during gastrointestinal surgery and to evaluate its performance, including feasibility for clinical application. Training data were created under the supervision of gastrointestinal surgeons. Test images and videos were randomly sampled and model performance was evaluated visually by 10 external gastrointestinal surgeons. The mean Dice coefficient of the 50 images was 0.46. The AI model could detect at least 75% of the loose connective tissue in 91.8% of the images (459/500 responses). False positives were found for 52.6% of the images, but most were not judged significant enough to affect surgical judgment. When comparing the surgeon’s annotation with the AI prediction image, 5 surgeons judged the AI image was closer to their own recognition. When viewing the AI video and raw video side-by-side, surgeons judged that in 99% of the AI videos, visualization was improved and stress levels were acceptable when viewing the AI prediction display. The AI model developed demonstrated performance at a level approaching that of a gastrointestinal surgeon. Such visualization of a safe dissectable layer may help to reduce intraoperative recognition errors and surgical complications.

**Supplementary Information:**

The online version contains supplementary material available at 10.1038/s41598-024-84044-5.

## Background

Surgical care has been improved through both academic and technological advances, but surgical complications remain a major problem^1–3^. According to a report on human performance errors in surgery, 30% of surgical adverse events were caused by errors in surgeons’ recognition^4^. In recent years, the supportive use of artificial intelligence (AI) in medicine has progressed remarkably, and various diagnostic support devices have been reported and commercialized^5–8^. These AI tools alone are not intended to provide a diagnosis but to support physicians in decision-making and performance of skills.

Technological solutions that support surgeons’ recognition skills are desirable for reducing complications in surgical care. AI medical devices that support surgeons in endoscopic or robotic surgery are still in their infancy and development is ongoing. Several AI models that recognize anatomical structures have been developed to support surgeons’ recognition^9–13^. Surgery relies on surgeons with many years of training to make accurate judgments and precise manipulations, and thus any AI tools that support them must demonstrate high performance. We have previously reported successful AI recognition of loose connective tissue (LCT)^14^. LCT is a critical anatomical landmark in abdominal surgery, where both functional preservation and oncological cure are important considerations^15–18^. However, our previous study^14^ evaluated the accuracy of LCT recognition in robotic gastrectomy only and the model did not show the performance required for clinical application.

We report here improvement of our previous AI model such that LCT is recognized and displayed as a layer safe to dissect in images captured during gastrectomy, colorectal surgery, and inguinal hernia surgery, which are common gastrointestinal and general surgical procedures. We report on the performance of the model, including its feasibility for clinical application.

## Methods

### Training dataset and development of the AI model

We enhanced our previously described AI model^14^ by training with more than 30,000 annotated portions of LCT fibers captured from 60 surgical videos of endoscopic surgeries performed at multiple Japanese hospitals from 2018 to 2022. These surgeries included 26 gastrectomies, 15 colorectal surgeries, 16 inguinal hernia repairs and 3 other surgeries. We used a web application provided by Incubit Inc. (Tokyo, Japan) for annotation process. Following a review of the training data used in our previous study^14^, the annotation of LCT fibers was conducted with greater precision and uniformity. Seven gastrointestinal surgeons and trained annotators under the surgeons’ supervision carefully annotated the LCT regions in the images to create the training data. We tuned the AI model based on the U-net and DeepLab v3. The AI model was then developed to automatically segment the LCT fibers.

### External evaluation committee and test dataset

To ensure fairness in the evaluation process, an independent external committee was established. The committee was composed of external gastrointestinal surgeons and a contract research organization which communicated with the external surgeons and oversaw the study. The external committee selected the video scenes and images for evaluation based on the criteria in this study.

To create the test dataset, 10 gastric, 10 colorectal, and 5 inguinal hernia surgeries were selected at random from a dataset of surgical procedures captured by two endoscopic imaging systems (VISERA ELITE II^®^, Olympus Inc., Tokyo, Japan and the da Vinci Xi Surgical System^®^, Intuitive Surgical Inc., Sunnyvale, CA) during surgeries performed at multiple hospitals in Japan in 2022. These test data were not included in the training data. From each of the 25 videos, two frames that clearly depicted LCT were extracted, yielding a total of 50 still images. We included evaluation images that clearly demonstrated the presence of LCT in the center of the images. Conversely, we excluded images in which the LCT was not clearly visible due to bleeding, smoking, or artifacts, and in which the LCT had degenerated due to inflammation or prior treatment. Additionally, we excluded evaluation scenes in which the surgical procedure was not progressing smoothly.

### The methods of the surgeon’s annotation

The surgeon’s annotation is an image that has been clearly delineated for the correct area of LCT through manual annotation, reflecting the surgeon’s recognition.

The external surgeons marked the outline of the LCT area on the raw images without confirming the AI prediction images. The research team faithfully painted the LCT fibers area according to the surgeons’ markings. Following the painting, the external surgeon checked the annotated area. If the external surgeon judged the revision of annotation to be necessary, the research team repainted. This process was repeated until no further revisions were necessary. After the annotation area was decided by an external surgeon, another surgeon checked it in the same way, and the same process was repeated. The surgeon’s annotation was finalized through manual annotation and with the approval of the external surgeons.

### The evaluation methods of the AI model performance

**Fig. 1:**
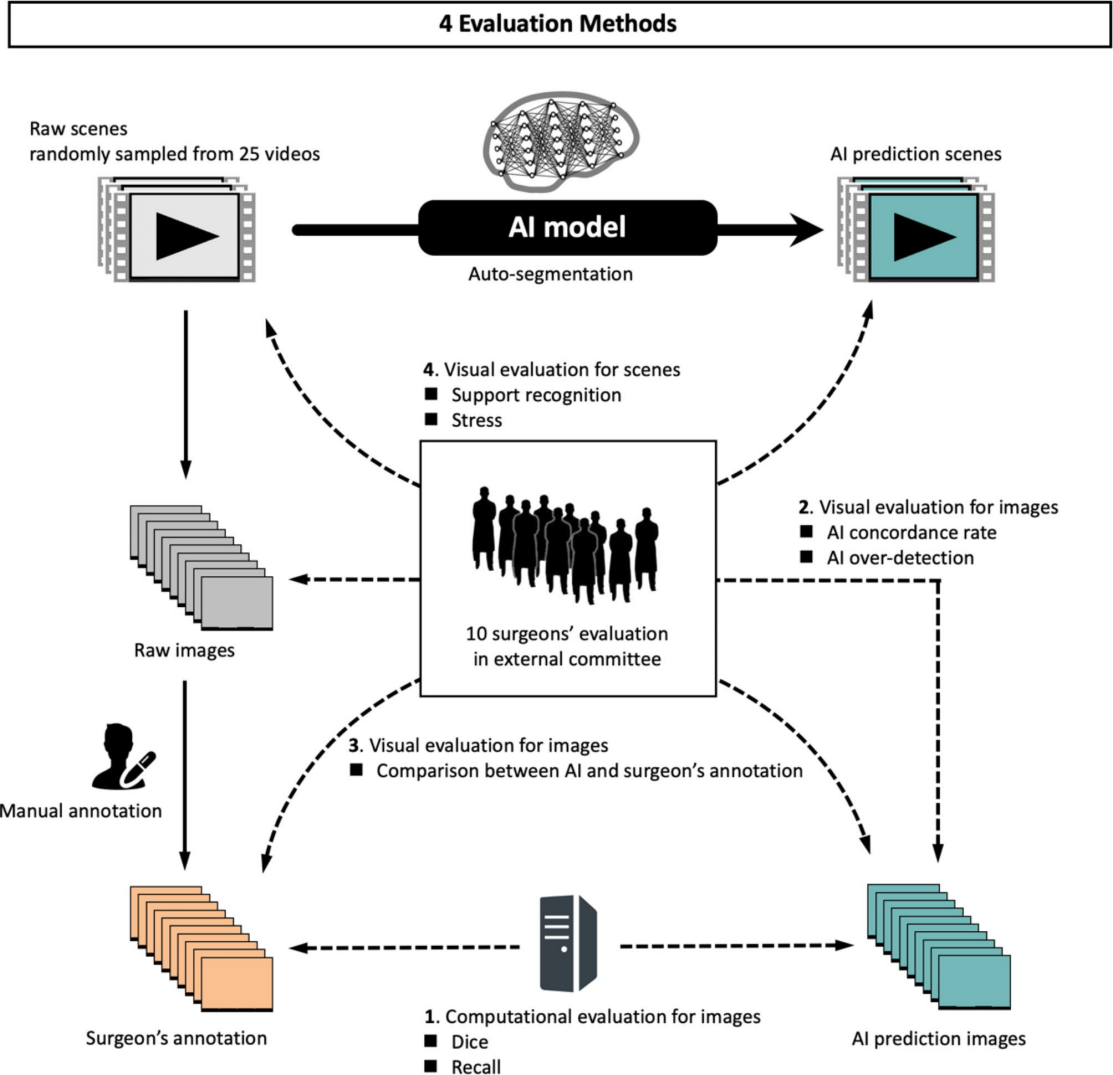
Four evaluation methods of the AI model.

The performance of the AI model was then evaluated by comparing the raw image, the AI prediction image, and the surgeon’s annotation using each of the following four methods (Fig. 1).

#### Quantitative computational evaluation of the still images

The surgeon’s annotation was determined by a consensus of three surgeons on the external evaluation committee. The similarity between the AI prediction image and the surgeon’s annotation was evaluated using the Dice coefficient, a performance metric widely used in medical image analysis for segmentation^19^. A cut-off value of 0.5 was set. Recall was utilized as an index for detecting LCT. The formulas for calculating the Dice coefficient and Recall are as follows, with higher values indicating better results.

Dice = $$\:\frac{TP}{TP+\frac{1}{2}(FP+FN)}$$, Recall = $$\:\frac{TP}{TP+FN}$$

Here, TP is true positive, FP is false positive, and FN is false negative.

#### Visual evaluation comparing the raw and AI prediction images

To help accurately evaluate the agreement between surgeons’ recognition and AI prediction, 10 external surgeons visually compared the raw images with the AI prediction images to determine the performance of the AI prediction. This approach was taken because of the complexity of determining and interpreting Dice coefficients as a measure of agreement in the clinical setting. Surgeons who did not participate in the annotation process conducted this visual evaluation. They evaluated the concordance rate of AI predictions and the nature of AI over-detection in the same 50 still images that were used in the computational evaluation.

The surgeons compared pairs of corresponding images consisting of two the raw image and the AI prediction image and answered the following questions on a 5-point scale: for the AI concordance score, “Does the AI recognize as LCT the same structures that you identified as LCT?”; and for AI over-detection, “If the AI recognizes structures as LCT that you identify as not being LCT, which of the following best describes the nature of this false positive?” (false positive but negligible impact; false positive but no impact on surgical judgment or procedure; false positive with minor impact on surgical judgment and procedure; and false positive with an unacceptable negative impact on surgery).

#### Visual evaluation comparing the surgeon’s annotation and AI prediction images

To compare the AI prediction with the surgeons’ recognition, an additional evaluation was performed with both the AI prediction image and the surgeon’s annotation. Two sets of manual annotation were created, one by the three external surgeons, corresponding to the surgeon’s annotation, and one by our research team independently. The 10 external surgeons on the evaluation committee selected the images that were closest and furthest from their own recognition while blinded to which of the 3 images was the AI prediction image. Half of 50 images were evaluated, after excluding obvious false positives that could easily be identified as AI predictions.

#### Visual evaluation of the video scenes

In a controlled laboratory setting, the 10 external surgeons were presented with two parallel displays, one showing the raw video and the other showing the AI prediction video. They were asked to assess the impact of viewing the AI analysis results in real-time on visualization and on their stress levels.

The test videos consisted of 10 randomly selected cases from a total of 25, each lasting 30 s. The surgeons carefully reviewed the raw and the AI prediction videos on the displays and answered the following questions on a 5-point scale: for the ability of AI to support recognition, “Does looking at the AI analysis display make it easier to recognize areas of LCT?”; and for their stress levels induced by viewing the AI prediction display, “Does the AI analysis display potentially cause you any stress due to false positives, false negatives, or superimposition on the display?”

The overall flow of the methods is shown in Fig. 1.

## Results

### Quantitative computational evaluation of the still images

The mean Dice coefficient was 0.46 (SD ± 0.10, range 0.16–0.62) and mean recall was 0.53 (SD ± 0.13, range 0.23–0.78). The change in accuracy was within 10% in the analysis by endoscopic system used and in that by surgical field (Table 1).

**Table 1:** The dice coefficients and recall of the 50 still images classified by endoscopic system and surgical field.

	Total	Endoscopic system		Surgical field		
		Visera Elite II	da Vinci Xi	Gastrectomy	Colorectal surgery	Hernia repair
Number of images/videos evaluated	50/25	30/15	20/10	20/10	20/10	10/5
Mean Dice (± SD)	0.46 (0.10)	0.47 (0.10)	0.44 (0.11)	0.43 (0.11)	0.48 (0.09)	0.45 (0.11)
Mean Recall (± SD)	0.53 (0.13)	0.55 (0.13)	0.50 (0.13)	0.56 (0.14)	0.52 (0.14)	0.49 (0.08)

SD, standard deviation.

### Visual evaluation comparing the raw and AI prediction images

The visual evaluation response rate was 100%. The mean AI concordance score was 4.62 (SD ± 0.67). As shown in Table 2, in 71.4% of cases, more than 90% of LCT was detected (score 5), and in 91.8% of cases, more than 75% of LCT was detected (score 4 or 5). The mean overall AI concordance score was 4.62. By surgical field, the scores were 4.65 for stomach, 4.62 for colon, and 4.59 for inguinal hernia. By endoscopic system, the scores were 4.66 for the VISERA ELITE II^®^ and 4.56 for the da Vinci Xi Surgical System^®^. In 47% of all images, there were no false positives. As shown in Table 3, in 96% of the images, surgeons answered that false positives from AI predictions would not impact surgery (score 3 or higher).

**Table 2:** AI concordance scores indicating the concordance rates between surgeons’ recognition and AI prediction.

Score	Concordance rates of AI prediction	Number (*N* = 500)	Proportion	Cumulative proportion
5	90% or more	357	71.4%	71.4%
4	Around 75%	102	20.4%	91.8%
3	Around 50%	37	7.4%	99.2%
2	Around 25%	3	0.6%	99.8%
1	10% or less	1	0.2%	100%

**Table 3:** AI over-detection score indicating the impact of false positives in AI prediction on surgeons’ recognition.

Score	Type of false positivity	Number (*N* = 500)	Proportion	Cumulative proportion
5	No false positives	237	47.4%	47.4%
4	False positives but negligible impact	155	31.0%	78.4%
3	False positives but no impact on surgical judgment or procedure	90	18.0%	96.4%
2	False positives with minor impact on surgical judgment and procedure	14	2.8%	99.2%
1	False positives with an unacceptable negative impact on surgery	4	0.8%	100%

**Fig. 2:**
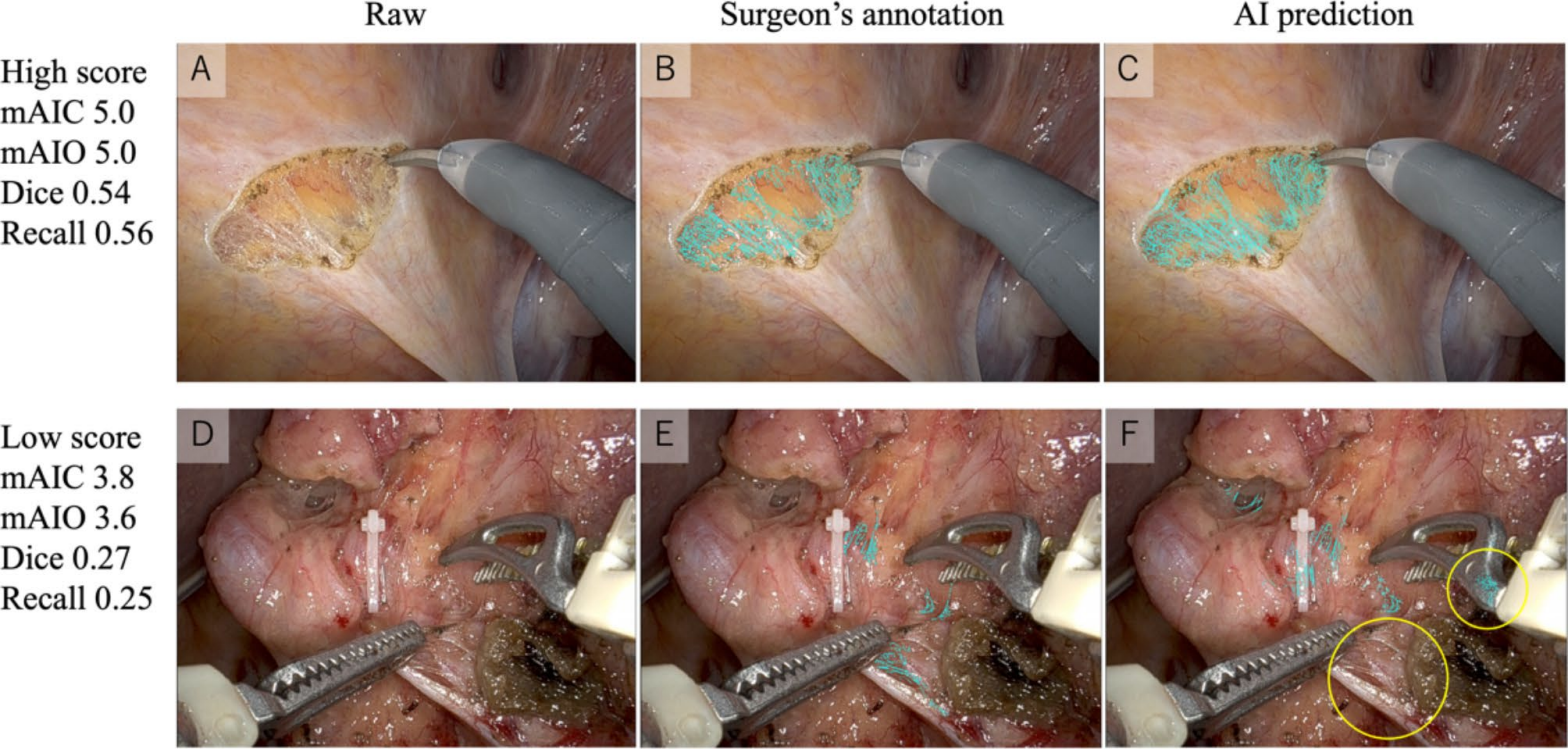
Images with a high (**A**–**C**) and low performance score (**D**–**F**). In the AI prediction image F, the yellow circle indicates a false positive for the surgical forceps as well as a false negative. mAIC, mean AI concordance score; mAIO, mean AI over-detection score.

Figure 2 shows a representative raw image, the surgeon’s annotation, and the AI prediction image with high or low performance scores. The AI prediction image with high scores accurately detected LCT and is difficult to distinguish from the surgeon’s annotation. In contrast, the AI prediction image with low scores contained both a false positive and a false negative. However, all surgeons answered that the false positives for surgical instruments would not have a negative impact on surgery (score 3, 4, or 5).

### Visual evaluation comparing the surgeon’s annotation and AI prediction images

The evaluation comparing the surgeon’s annotation with the AI prediction image focused on the image that was closest or furthest from the surgeon’s own recognition out of the 25 images provided. The surgeons were instructed not to respond to images that were difficult to evaluate. Table 4 shows that 5 of the 10 surgeons found the AI prediction images to be closer to their own recognition, while 7 surgeons found them to be the furthest. To ascertain the veracity of the training data, a comparative analysis was performed between the surgeon’s annotation created by our research team, who also created the training data, and the surgeon’s annotation created by the external surgeons. The surgeon’s annotation created by our research team had a lower rate of images that were both closest to and furthest from the surgeons’ recognition. It was thus challenging to determine which image was superior.

**Table 4:** Evaluation of images closest to or furthest from the recognition of the 10 surgeon evaluators.

Evaluator	Closest to your recognition			Furthest from your recognition		
	Manual annotation		AI prediction	Manual annotation		AI prediction
	Internal	External		Internal	External	
Surgeon 1	*9*	5	6	2	3	*9*
Surgeon 2	3	6	*9*	0	3	*7*
Surgeon 3	5	2	*14*	6	*7*	3
Surgeon 4	3	9	*10*	9	4	*9*
Surgeon 5	5	*11*	8	5	4	*16*
Surgeon 6	8	*10*	6	5	2	*11*
Surgeon 7	6	2	*12*	3	*12*	8
Surgeon 8	7	*14*	1	3	1	*16*
Surgeon 9	2	3	*19*	6	*10*	3
Surgeon 10	10	*12*	3	0	5	*20*
Total	1/10	4/10	5/10	0/10	3/10	7/10

Underlined numbers indicate the most votes among the 25 images.

### Visual evaluation of the video scenes

Table 5 shows that the surgeons judged that AI support helped with LCT recognition in 99% of cases (score 2 or higher). Table 6 shows that in 57% of cases, viewing the AI-monitor caused no stress (score 5), while in 99% of cases, it caused an acceptable level of stress (score 3 or higher).

**Table 5:** Ability of the AI to support surgeon’s recognition of LCT.

Score	Visualization of LCT with AI	Number (*N* = 100)	Proportion	Cumulative proportion
5	Extremely easy to recognize	47	47.0%	47.0%
4	Much easier to recognize	35	35.0%	82.0%
3	Easier to recognize	12	12.0%	94.0%
2	Slightly easier to recognize	5	5.0%	99.0%
1	Not easy to recognize	1	1.0%	100.0%

LCT, loose connective tissue.

**Table 6:** Stress score caused by viewing the AI prediction display.

Score	Stress caused by AI prediction displayed on a sub-monitor	Number (*N* = 100)	Proportion	Cumulative proportion
5	No stress	57	57.0%	57.0%
4	Some stress, but it does not affect using the AI (can be used adequately as reference information)	37	37.0%	94.0%
3	Stress is present, but using the AI is acceptable (is acceptable for display on a sub-monitor)	5	5.0%	99.0%
2	Stress is high and using the AI is undesirable (even if a sub-monitor is available, the user does not look at it; their focus is on the main monitor)	1	1.0%	100.0%
1	Stress is extremely high and using the AI is unacceptable (is unacceptable even for display on a sub-monitor)	0	0.0%	100.0%

Video 1 (Supplementary information) shows the raw video and the AI prediction video side-by-side. In inguinal hernia repair, rectal resection and gastrectomy, the AI recognizes LCT and highlights it with cyan. The AI accurately displays delicate LCT fibers in inguinal hernia repair with high score of visual evaluation. Although some clear false positives for the surgical device are apparent in rectal resection, we do not believe that it would negatively affect the surgeon’s judgment. In one of the scene in gastrectomy, the score of visual evaluation was relatively low.

## Discussion

In this study, gastrointestinal surgeons visually evaluated the performance of our latest AI model that recognizes LCT and determined that it was able to detect at least 75% of the LCT in 91.8% of the images evaluated. False positives were found in 52.6% of the images. However, most were not significant enough to affect surgical judgment or manipulation. The false positives were located mainly in areas with surgical instruments or where LCT was clearly not present and did not cause the surgeon confusion. In addition, when the AI prediction images were compared with the surgeon’s annotation, the AI prediction images had comparable accuracy. Thus, our AI model achieved a level of accuracy comparable to that of experienced gastrointestinal surgeons when the images were limited to those without obvious false positives.

In the quantitative evaluation, the overall mean Dice coefficient was 0.46. Analysis by endoscopic system or surgical field revealed that the Dice coefficient was similar. The AI model, which included training data from the stomach, colon, and inguinal hernia and from two types of surgical endoscopic systems, showed consistent accuracy. Several studies have investigated machine learning for recognizing anatomical structures. Most of these studies used the Dice coefficient or IoU (Intersection over Union) to evaluate the performance of AI models^9,10,13,20^. However, the accuracy of these quantitative evaluation methods varies depending on the number of pixels in the correct area for the target structure and on the methods used to create the ground truth. Therefore, interpreting whether an anatomical structure is accurately reflected based solely on quantitative values can be challenging^21^. The Dice coefficient tends to be lower when the correct area of the target has fewer pixels. The present study also found a positive correlation between the number of pixels in the surgeon’s annotation and the Dice coefficient (data not shown).

To overcome this limitation, in our method surgeons visually inspect and compare the AI prediction with their own recognition. By comparing the AI prediction image with the reference annotation determined by the surgeons, we have demonstrated that the AI was able to recognize LCT with an accuracy comparable to that of surgeons. Furthermore, no superiority was found between the reference annotation determined by the research team and that determined by the external surgeons, indicating the accuracy of the training data used.

We also conducted verification using videos in a simulated environment similar to a real-world environment. Visualization and stress levels were evaluated to determine the effectiveness and safety of using the AI developed. Most of the surgeons judged that visualization of LCT was improved and that there was either no stress or an acceptable level of stress with its use. These results suggest that the present AI model can predict LCT with an accuracy comparable to that of an experienced surgeon and can produce visibility-enhancing effects without inducing excessive stress.

There are two expected benefits of our AI model: one is the prevention of misrecognition, and the other is intraoperative educational support. Even for expert surgeons who can easily recognize anatomical structures, their physical and mental condition during surgeries can affect their cognitive abilities and sometimes affect their judgment. In a study on inattentional blindness, where an object can be visually perceived but not recognized if attention is not paid to it^22^, 24 radiologists participated in an experiment to detect pulmonary nodules on chest computed tomography scans. When a gorilla that was 48 times larger than the average lung nodule was embedded in the images without informing the radiologists, 83% of the radiologists did not recognize the gorilla, despite 60% of them actually viewing the area containing the gorilla, as confirmed by eye-tracking. These findings indicate that even experts are subject to inattentional blindness. It is hoped that AI-supported awareness, such as that offered by our AI model, can be one means to prevent recognition errors such as inattentional blindness during surgery. Experienced surgeons will have had the experience of being able to recognize something that they could not recognize before, either through training or through awareness. The use of AI, which can color anatomical structures, has the potential to accelerate cognitive mastery among trainee surgeons.

This study has several limitations. First, at the time of this study, the AI model was an unapproved medical device and was used for research purposes only. It is important to note that the effects of recognition support and stress were evaluated in a simulated environment, not during actual surgery. Second, we excluded images from the test dataset of hemorrhage that did not clearly show LCT as well as structures that showed degeneration due to inflammation or prior treatment. We believe that further improvement and verification is needed for our AI to be applied to atypical and highly challenging surgeries. Third, the evaluation comparing the AI prediction images with the surgeon’s annotation was conducted after first excluding AI prediction images with obvious false positives. It is important to note that the AI-generated false positives might contain errors that humans would not make, although the nature of those seen in this study would not have a strong negative impact on surgery.

The clinical application of this AI model has the potential to support safer surgery by providing a visual representation of LCT that highlights the dissectable layers, using color-coded displays. In the future, we plan to apply this AI model in clinical practice to verify its impact on surgical outcomes.

## Conclusion

The AI developed was able to recognize LCT in images obtained from gastric, colorectal, and inguinal hernia surgeries with an accuracy approaching that of a gastrointestinal surgeon. Such visualization of LCT, which constitutes a dissectable layer, may contribute to reducing intraoperative recognition errors and surgical complications.

## Electronic supplementary material

Below is the link to the electronic supplementary material.

Supplementary Material 1

## Data Availability

We cannot share the raw data and materials because the ethics committees of the participating medical institutions prohibit the publication of raw data including patients’ clinical data. However, the analyzed dataset is available from the corresponding author on reasonable request and with the permission of the participating medical institutions.
